# Rapid optical determination of salivary cortisol responses in individuals undergoing physiological and psychological stress

**DOI:** 10.1038/s41598-024-69466-5

**Published:** 2024-12-30

**Authors:** Tashfia Ahmed, Michael B. Powner, Meha Qassem, Panayiotis A. Kyriacou

**Affiliations:** 1 Research Centre for Biomedical Engineering (RCBE), School of Science and Technology, City, University of London, Northampton Square, London, EC1V 0HB UK; 2 Centre for Applied Vision Research, School of Health and Psychological Sciences, City, University of London, Northampton Square, London, EC1V 0HB UK

**Keywords:** Biomedical engineering, Predictive markers, Predictive markers

## Abstract

Traditional methods for management of mental illnesses in the post-pandemic setting can be inaccessible for many individuals due to a multitude of reasons, including financial stresses and anxieties surrounding face-to-face interventions. The use of a point-of-care tool for self-management of stress levels and mental health status is the natural trajectory towards creating solutions for one of the primary contributors to the global burden of disease. Notably, cortisol is the main stress hormone and a key logical indicator of hypothalamic-pituitary adrenal (HPA) axis activity that governs the activation of the human stress system. Therefore, the measurement of cortisol is imperative to lead the discussion of the relationship between psychological stress and mental health deterioration. The aim of the current study was to determine salivary cortisol concentrations of healthy individuals undergoing the MAST protocol for human stress activation, through optical-colorimetric techniques. The study demonstrates the use of the blue tetrazolium (BT) method as successful means of rapid measurement of cortisol in saliva, comparable to the gold-standard technique i.e., enzyme-linked immunoassays (ELISA) with a coefficient of determination (R^2^) of 0.997. The results support the future development of a point-of-care optical sensor-based device and mobile application for cortisol monitoring and stress profiling in adults.

## Introduction

The relationship between persistent psychological stress and mental health complicacies has been one of the focal points of psychological stress research in the recent years^1,2^. The dysfunctionality of the hypothalamic–pituitary–adrenal (HPA) axis, which regulates the stress response, often results in chronic stress and has proven to lead to the deterioration of mental health^3,4^. Therefore, it is of utmost significance to fully comprehend the effects of chronic stress on the human body and brain, and quantify stress responses, towards management and prevention of mental health deterioration.

Cortisol is a glucocorticoid which is considered the key logical indicator of stress. In previous studies, elevated cortisol secretion has been correlated with higher severity of negative symptoms associated with mental health. As one of the governing hormones of the stress response, cortisol is considered the ‘hormonal endpoint’ of the HPA axis. Once secreted, the glucocorticoid facilitates the body’s recovery from a stressor. The significance of cortisol level dynamics on the symptoms of mental illnesses showcases that cortisol is a critical factor in the development of several mental illnesses, especially those involving depressive and negative symptoms^5^. Therefore, cortisol is an ideal biomarker for stress monitoring and management, due to its primary contributing factor to the development of chronic stress and in some cases, clinical depression, via endocrinal means. Regular levels of salivary cortisol range from 0.7 to 11 ng/mL^6,7^. Salivary cortisol levels are superimposed on the circadian rhythm, with morning cortisol levels reaching as high as 27.3 ng/mL before declining throughout the day^6,7^. This independent phenomenon is known as the cortisol awakening response, comprising of an increase in cortisol peak values by at least 50% within 30 min post-awakening^8,9^.

Several methods have been explored to determine cortisol concentration in serum and saliva for evaluation of psychological stress, including LC–MS/MS, immunoassays and ELISAs, which is considered the gold standard analytical technique for serum and saliva cortisol determination. Although these methods are the most used for determining salivary cortisol, they may not be appropriate in low-cost and time-dependent setting where rapid cost-effective analysis would be favoured, as they require skilled personnel and highly specialised equipment^10^. Furthermore, the invasive nature of blood tests is also undesirable in the case of frequent appointments. Thus, the need for a rapid and accessible method to determine cortisol levels is of utmost significance, as mental health prevalence is unprecedented globally. Various studies have noted the relationship between salivary cortisol and psychological stress, more so than serum cortisol due to the binding properties of cortisol in the bloodstream^11–13^. Therefore, the measurement of salivary cortisol is an established method for determining psychological stress noninvasively, albeit through enzymatic methods such as ELISA. Tu et al.^14^ reported the use of various chromogenic agents for the determination of cortisol concentration through a series of in vitro experiments, and a pilot study using human sweat samples. The present study utilised one of the noted chromogenic agents (blue tetrazolium) for the determination of cortisol in human saliva samples, taken from participants who underwent a modified version of the Maastricht Acute Stress Test (MAST)^15^. The MAST has been found to yield superior salivary cortisol responses in a rapid and non-invasive manner for effective stress elicitation^15^. Evidently, Tu et al.^14^ concluded that the BT method is comparable to the gold-standard ELISA technique due to the rapid reaction rate, low limit of detection (LoD) and greater range of detection. Presently, the colorimetric determination of salivary cortisol concentration was observed through optical spectroscopy in the visible region (400–800 nm). This method facilitates the rapid and accurate detection of salivary cortisol concentrations, which strongly correlated with cortisol ELISAs i.e., the gold standard. This study is fundamental towards the development of a point-of-care device for rapid measurement of salivary cortisol levels in stressed individuals. The current study highlights the robust BT method for the optical determination of salivary cortisol, offering several advantages such as rapid result output, cost-effectiveness and ease of use compared to the existing gold-standard, the ELISA.

## Materials and methods

### Participants

A total of 22 healthy adults with a mean age of 28.18 ± 9.00 years participated in the current study, including 12 males and 10 females. 3 participants from this cohort were excluded from this study due to insufficient saliva samples. The participant information sheet was provided to interested volunteers and eligibility was assessed using a screening questionnaire. Exclusion criteria was adopted from Smeets et al., which included diagnosed mental illnesses, endocrine disorders, cardiovascular diseases, pregnancy, oral infections, heavy smoking (> 10 cigarettes/day), recreational drug use and excessive alcohol consumption (> 14 units/week)^15,16^. Written informed consent was provided by all participants. This study was carried out under approval by the City, University of London, School of Science and Technology Senate Research Ethics Committee. Participants were informed to refrain from caffeinated drinks at least 3 h prior to the MAST and abstain from food or drink consumption 1 h prior to the test. This was implemented to ensure that the results of the salivary analysis were not influenced by recent food or drink intake. Furthermore, the protocol was conducted at approximately similar times (between 12 and 4 pm) for all participants to ensure the effects of the circadian rhythm of cortisol responses did not interfere with the study’s findings. All methods were performed in accordance with the regulations and guidelines under the approval of the City, University of London, School of Science and Technology Senate Research Ethics Committee, and in accordance with the Declaration of Helsinki.

### Maastricht acute stress test protocol

Upon informed consent, participants were informed that the stress test would involve alternating trials between hand immersion (HI) and mental arithmetic (MA) tasks of randomly determined durations. Participants were unaware of the number and duration of trials. The HI task involved immersion of the participant’s non-dominant hand into ice-cold water (2 °C). The MA task involved counting aloud backwards from 2043 in steps of 17. Participants were informed that mistakes with accuracy or slow response (> 5 s) would lead to negative feedback from the experimenter and restarting of the MA task. The duration of all trials was pre-determined, and the same pattern was used for all participants. After the MAST was completed, the participant was debriefed and given instructions for the intervention period. The intervention period consisted of a 20 min relaxation phase where the participant was advised to relax in the monitoring room, unmonitored by the experimenter.

### Physiological measures

Salivary cortisol was measured in response to the MAST as a measure of stress activity. For each participant, a total of four saliva samples were collected via the Cortisol Salivette (Cortisol Salivette^®^, SARSTEDT, Numbrecht, Germany). For sample collection, the Salivette^®^ swab is placed in the mouth for 2 min without chewing. A baseline saliva measurement was taken prior to the MAST (T-Base), immediately after MAST completion (T-0), 20 min after completion (T-20) and post-relaxation (T-Relax). After collection, the swab is placed into the Salivette Cortisol tube for centrifugation at 1000 g for 2 min at room temperature, using the Biofuge Primo R (Sorvall, Waltham, MA, USA). The average saliva volume that was recovered was 1.8 ± 0.3 ml. Centrifuged samples were stored at − 80 °C before subsequent analysis. Cortisol levels were determined spectroscopically as well as with commercially available ELISA kits (Parameter Cortisol Assay, R&D Systems, Minneapolis, MN, USA).

### Subjective measures

The NHS depression and anxiety self-assessment quiz was administered as a baseline measurement of participant mental health state, prior to starting the MAST^17^. The quiz consists of 18 questions based on the Personal Health Questionnaire (PHQ-9) and the Generalised Anxiety Disorder Scale (GAD-7) developed by Kroenke and Spitzer et al. as methods to assess and monitor depression and anxiety severities, respectively^18,19^. Based on the responses from the assessment, participants were given a depression score from 1 to 21 and an anxiety score from 1 to 21, with increasing scores correlating with increased severity of depression and anxiety symptoms. To ensure the safety of all participants, those scoring above 12 were advised to withdraw from the study, as approved by the Senate Research Ethics Committee, City, University of London. Immediately after the MAST protocol, participants were asked to rate their experiences of stress on a 5-point Likert scale, ranging from 1 to 5 (1 = not stressful at all, 5 = extremely stressful).

### Reagents

3,3′-3,3′-dimethoxy-4,4′-biphenylene bis 2,5-diphenyl-2 h-tetrazolium chloride (blue tetrazolium), Tetramethylammonium hydroxide, 25 wt.% in methanol, and methanol (> 99.7%) were acquired from Fisher Scientific (Fisher Scientific, Waltham, MA, USA). For the preparation of the dye, 1.2 g of blue tetrazolium was dissolved in 200 ml of methanol. Additionally, a 1% v/v tetramethylammonium hydroxide solution was prepared by diluting 5 ml of tetramethylammonium hydroxide in 45 ml of methanol. As stated by Tu et al.^14^, methanol decreases the reaction time by 25%, with no changes in sensitivity. Therefore, analytical grade methanol (> 99.7%) was used as the primary solvent for reagent preparation.

### Optical spectroscopy measures

To determine cortisol concentration in saliva samples through optical spectroscopy, 50 µl of the centrifuged saliva was combined with equal volumes (200 µl) of blue tetrazolium dye solution and tetramethylammonium hydroxide solution (fourfold sample dilution). The BT method was experimentally validated for the visual inspection of cortisol concentration by Tu et al.^14^ where the characteristic absorption peak of the colorimetric reaction was 510 nm. The visual colour change which includes a 10 min development period, from translucent yellow to magenta, is stable for up to 12 h. Therefore, measurements can be made at any time within this 12 h window. The intensity of the magenta colour increases with rising concentrations of cortisol; therefore, the determination of salivary cortisol at high levels is detectable by the naked eye. The peak absorption rises with respect to increasing salivary cortisol concentrations, showcasing a linear relationship. The colorimetric reaction is dependent on the hydrolysis rate of cyclic diacetyl in the blue tetrazolium dye, whereby the cortisol concentration determines the rate of full colour development in the sample. The cortisol concentration from each sample was reported in ng/mL after accounting for the dilution factor (fourfold sample dilution). The limit of detection (LoD) for the BT method is 0.7623 ng/mL, whilst the LoD for the gold-standard ELISA is 0.2 ng/mL. The upper limits of detection for the proposed BT method is 36 ng/mL, which is well beyond the normal salivary cortisol range presented in humans^7^.

Notably, the colorimetric reaction is stabilised in solvents with a large dielectric constant e.g., water, which indicated the suitability of measuring cortisol levels in saliva via the BT method, as the composition of saliva is 99% water. Hence, the redox reaction which leads to the colour development as a function of cortisol concentration was decelerated to a discernible level, that could be detected by the spectrometer. 450 µl of the prepared sample was pipetted into plastic Eppendorf disposable UV–Vis transparent cuvettes, with a spectral range between 220 and 1600 nm and path length of 10 mm. Three consecutive absorbance spectra were acquired for each prepared sample with the use of a dual beam spectrophotometer (Lambda 1050) in the spectral region between 450 and 650 nm (PerkinElmer Corp, Waltham, MA). The spectrophotometer was configured in a 3-cycle format, to yield three spectra from each sample, within the specified spectral region, at a step interval of 1 nm. The Lambda 1050 was equipped with the 3-detector module. Reference and sample attenuators were set to 100% and the reference cuvette was kept blank for the entire duration of the protocol. Baseline correction of 100% transmittance/0% absorption was taken to account for the effects of instrument and ambient environmental noise.

### ELISA

Cortisol ELISA kits (Parameter Cortisol Assay, R&D Systems, Minneapolis, MN, USA) were used according to manufacturer’s recommended protocol with a fivefold saliva sample dilution, to determine salivary cortisol levels. 50 µl of saliva sample was mixed with 200 µl of diluent. Cortisol concentrations were reported in ng/mL after considering the sample dilution. All samples were tested in duplicate, and a cortisol standard curve was included on each assay plate.

### Data analysis

#### Spectroscopy and regression analysis

Spectra collection was carried out using UVWinLab for the Lambda 1050 spectrophotometer. Spectral pre-processing and analysis were performed in MATLAB R2020b, (MathWorksTM, Natick, MA, USA), with use of the Statistics and Machine Learning Toolbox, on two datasets of samples to determine salivary cortisol concentrations in participants undergoing the MAST protocol. The datasets were divided according to sample placement on each ELISA plate, for simpler comparison and validation. 3 spectra were acquired from each sample, which were pre-processed through baseline subtractions, averaging, and smoothing. Savitsky-Golay smoothing was implemented (interval:5, polynomial order:2), as well as 2nd derivative calculations, to reduce noise and improve discernibility between samples through enhanced spectral features. One dataset comprising of samples of known cortisol concentrations in artificial saliva was used to develop a standard curve, which was later implemented in a linear regression curve to create a prediction model. The range of cortisol concentrations used as a standard set can be found in Table 1. Peak absorbance values at 510 nm from each spectrum were used to predict concentration values against the prediction model. This was compared against the results obtained from the cortisol ELISA protocol using the Pearson’s correlation coefficient.

**Table 1:** Calibration set of known concentration cortisol-spiked artificial saliva samples and representative absorbance values at 510 nm, for use in predictive regression model for cortisol determination in human saliva samples.

Artificial salivary cortisol concentrations (ng/mL)	Absorbance value
0.5	0.268
5.5	0.398
11	0.500
16	0.633
21.5	0.767
27	0.866
32.5	0.969

#### ELISA

Optical density results were obtained from each sample through the cortisol ELISA protocol. Pre-processing of this data was conducted in Excel, which involved averaging duplicates and subtracting the B0 value from each plate respectively. The results were then analysed as recommended in the manufacturer’s instructions, using a 4-PL (Parameter Logistic) regression model, Quest Graph™ Four Parameter Logistic (4PL) Curve Calculator (AAT Bioquest Inc., Pleasanton, CA, USA). A standard curve was developed using the non-linear regression model which was then implemented to determine the cortisol concentrations of all samples within the two datasets with a total of 19 participants i.e., 76 samples. The cortisol concentrations for each sample were reported in ng/mL, after accounting for the dilution factor (fivefold sample dilution).

## Results

### Analytical performance of BT protocol for salivary cortisol concentration determination

**Figure 1:**
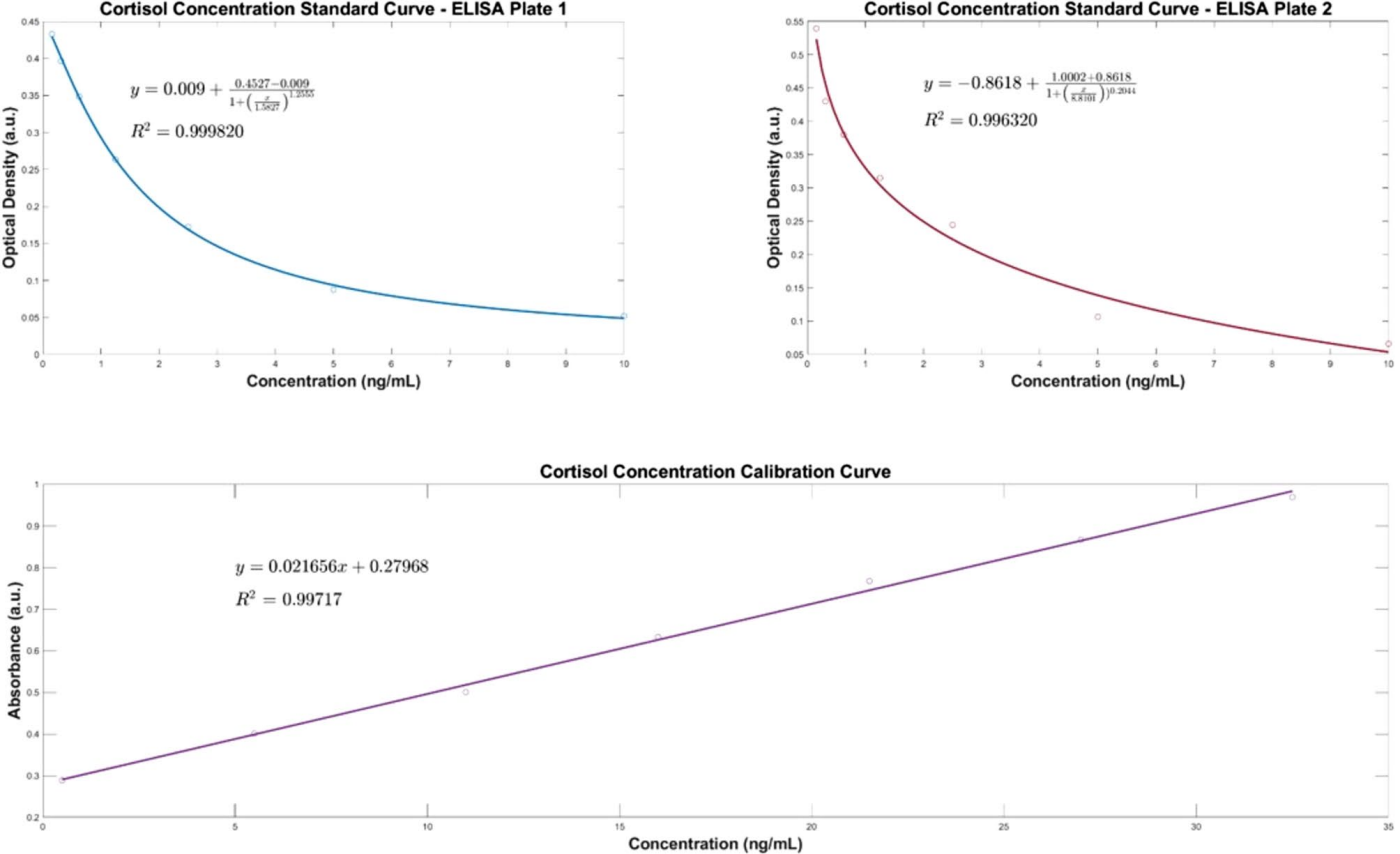
Analytical performance comparison of the ELISA protocol (top left and right) and Blue Tetrazolium (BT) method (bottom) for monitoring salivary cortisol in clinical range (ng/mL) using linear regression analysis. Both methods show high sensitivity for determination of cortisol levels within human saliva samples.

**Figure 2:**
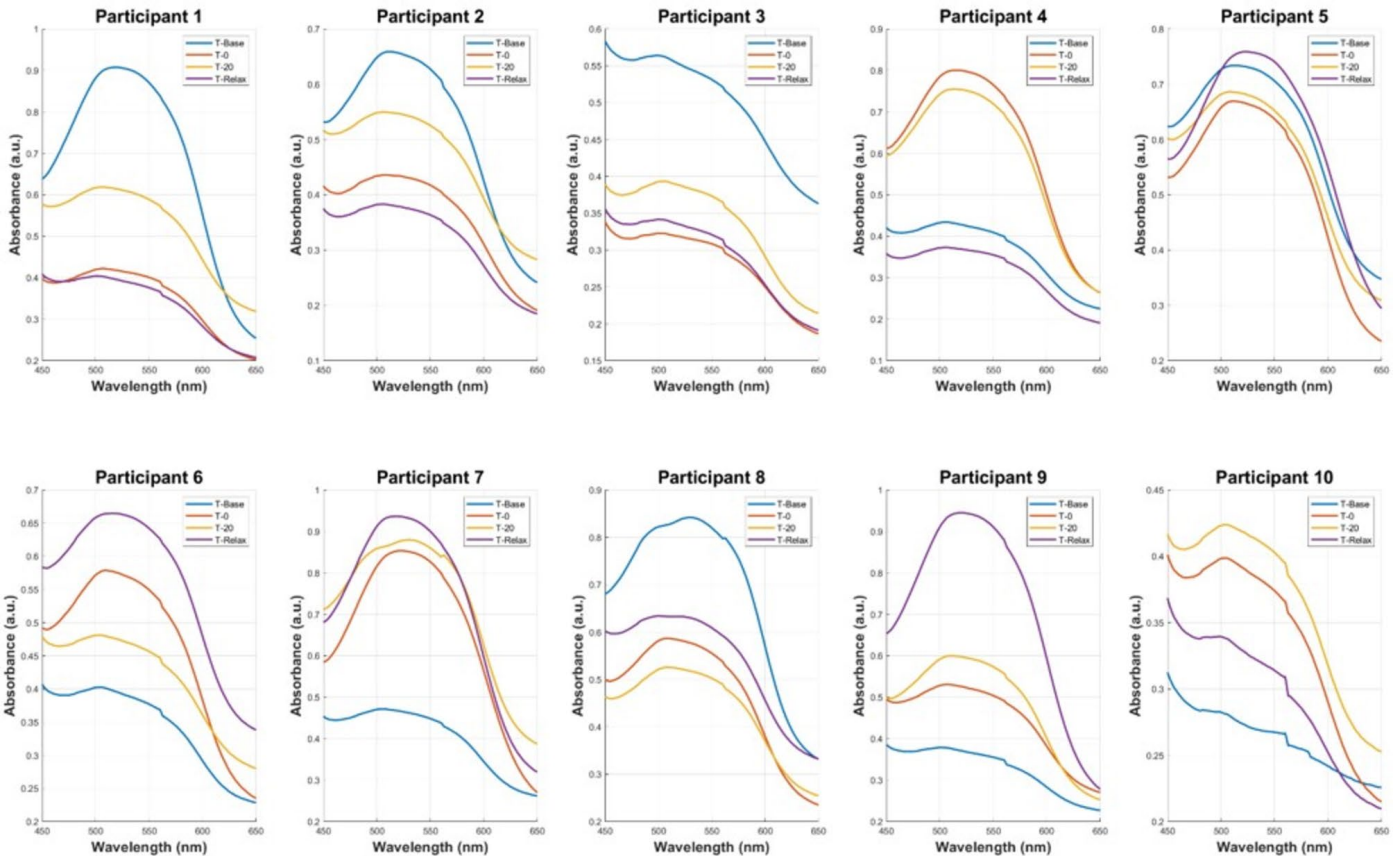
Absorbance spectra of cortisol in blue tetrazolium and tetramethylammonium hydroxide reagents with characteristic peak at 510 nm, dataset 1 (Participants 1–10), including post-processing (averaging, baseline subtractions and Savitsky-Golay smoothing). Peak separation between varying concentrations shows clear distinction between detected cortisol concentrations.

**Figure 3:**
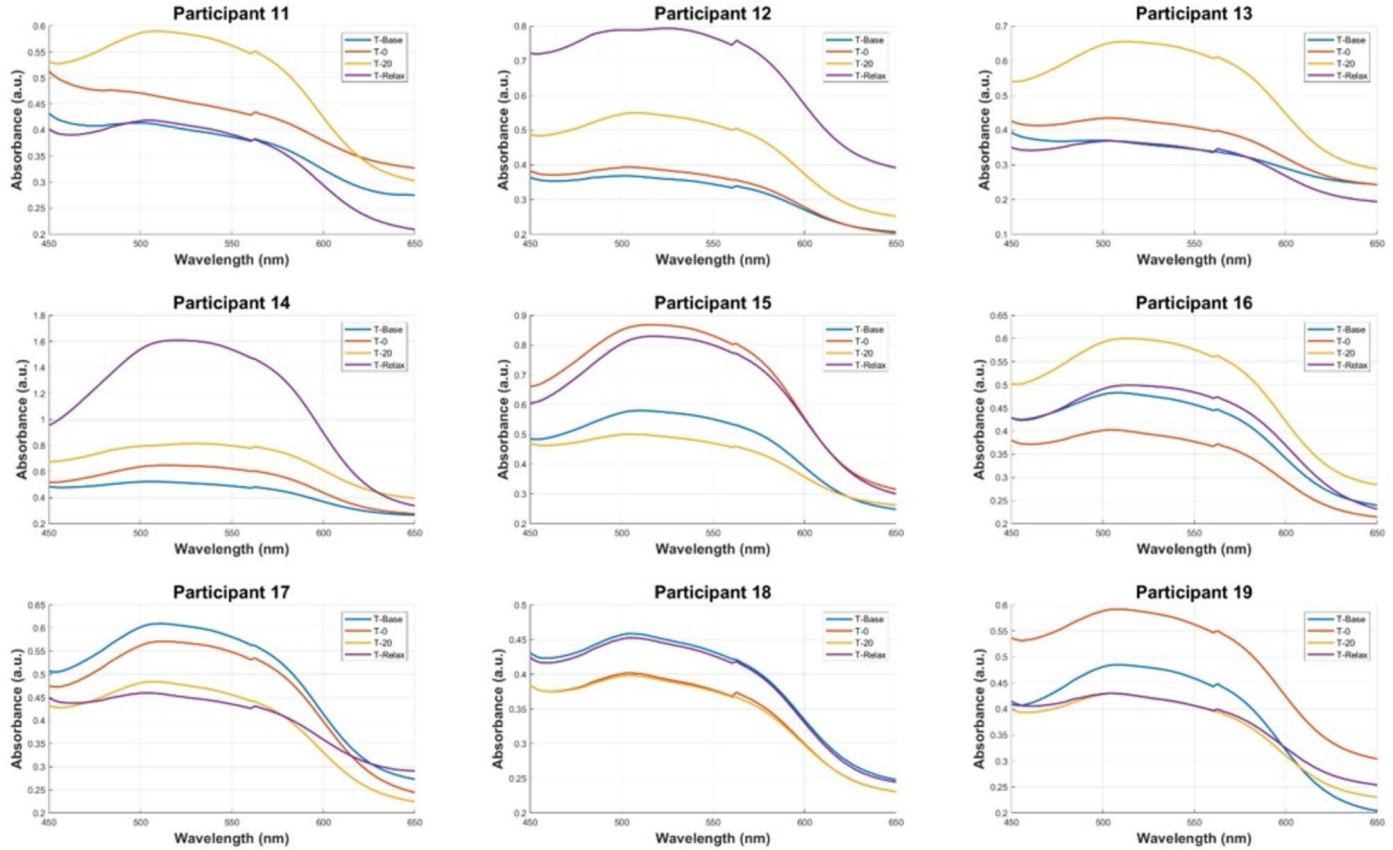
Absorbance spectra of cortisol in blue tetrazolium and tetramethylammonium hydroxide reagents with characteristic peak at 510 nm, dataset 2 (Participants 11–19), including post-processing (averaging, baseline subtractions and Savitsky-Golay smoothing. Peak separation between varying concentrations show clear distinction between detected cortisol concentrations.

**Figure 4:**
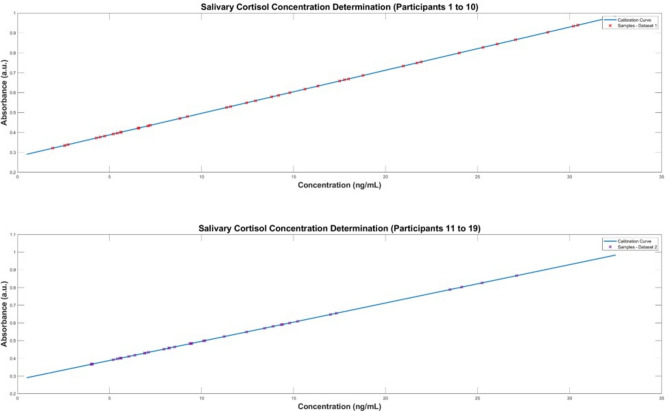
Cortisol determination using the salivary cortisol concentration standard curve at 510 nm from BT method on dataset 1—Participants 1–10 (top) and dataset 2—Participants 11–19 (bottom).

**Figure 5:**
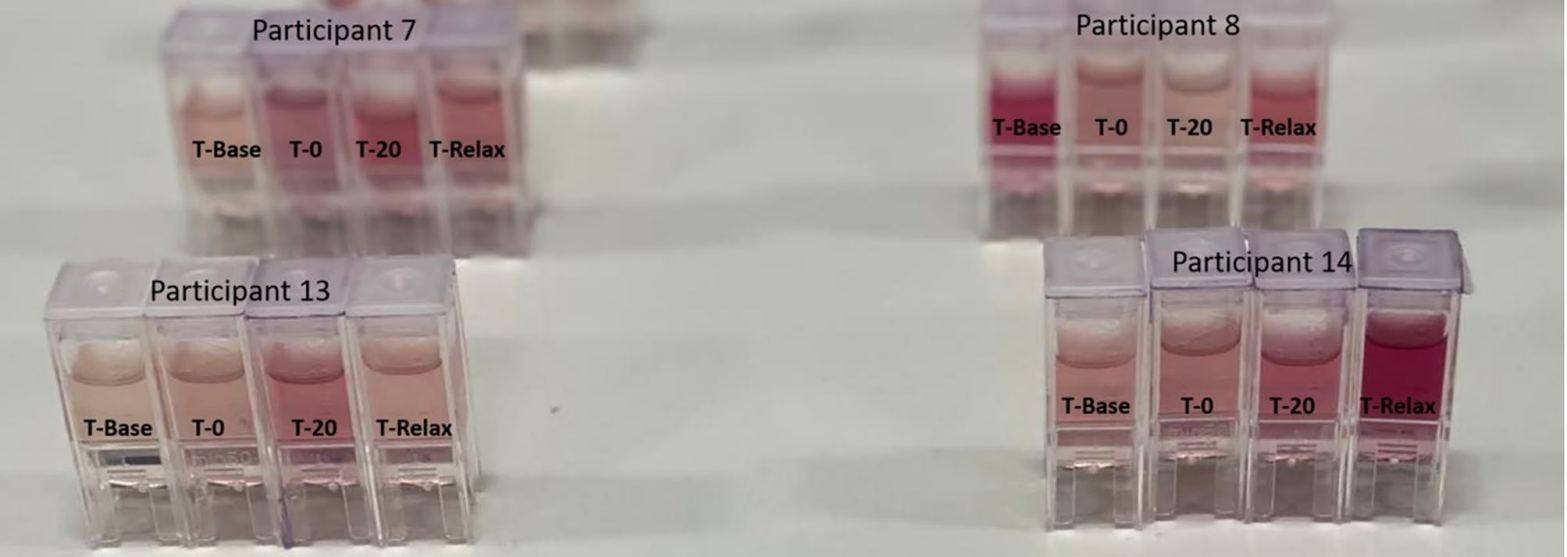
Optical images of colour change with varying concentrations of salivary cortisol in participants 7 (top left), participant 8 (top right), participant 13 (bottom left) and participant 14 (bottom right) via BT method.

**Figure 6:**
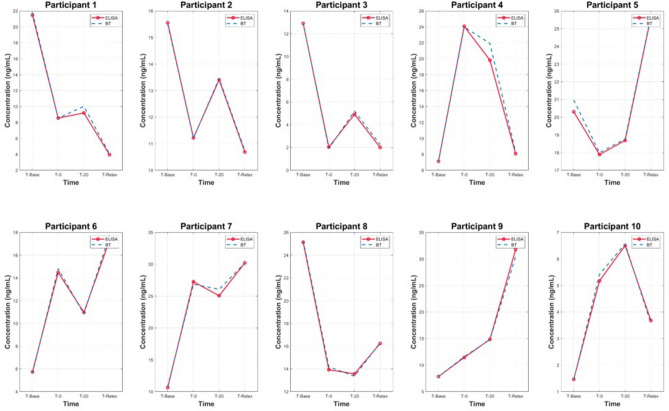
Salivary cortisol monitoring via BT method (blue) and ELISA method (red) for dataset 1 (participants 1–10) undergoing the MAST protocol. Significant correlation between the proposed experimental (BT) method and the gold standard method (ELISA).

**Figure 7:**
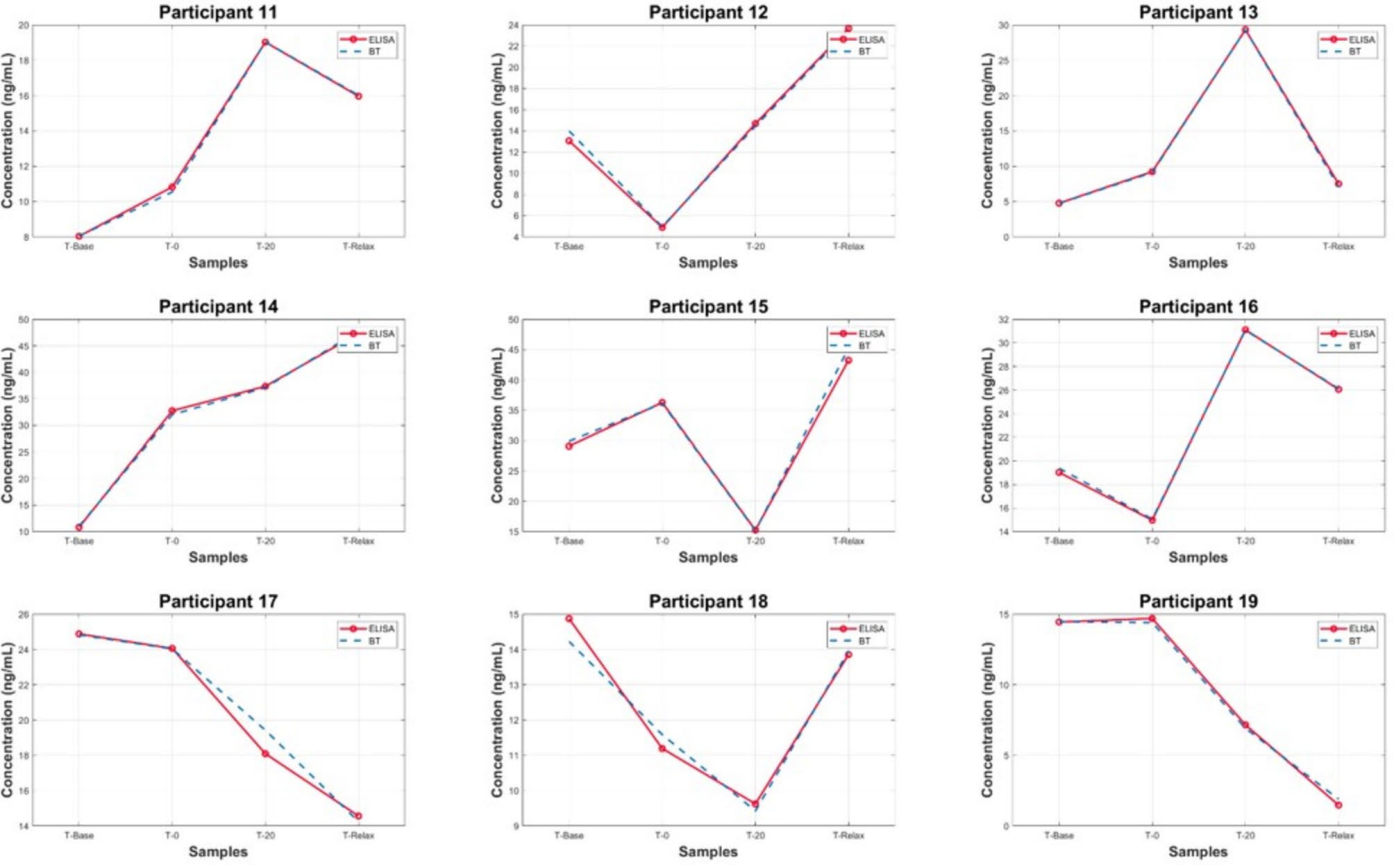
Salivary cortisol monitoring via BT method (blue) and ELISA method (red) for dataset 2 (participants 11–19) undergoing the MAST protocol. Significant correlation between the proposed experimental method (BT) and the gold standard measurement (ELISA).

The samples were separated into two datasets, to replicate the same order that was used in the cortisol ELISA protocol. A BT standard curve was determined with a standard set of known cortisol concentrations in artificial saliva samples which was then compared with the cortisol ELISA standard curves, shown in Fig. 1. The characteristic peak around 510 nm from the UV–VIS spectra of the standard set demonstrates the linear correlation between increasing cortisol concentration and absorbance. This linear relationship was used to predict salivary cortisol concentrations in the human saliva samples acquired from participants undergoing the MAST protocol. The Beer-Lambert Law was employed to determine the cortisol concentration from the samples. The law states that a linear relationship exists between the analyte concentration and the absorbance of the sample. A linear regression model (R^2^ = 0.997) was utilised to facilitate in the quantification of the target analyte concentration (cortisol) in the collected saliva samples. The pre-processed spectra from the in-vivo trial are visualised in Figs. 2 and 3. The results demonstrate the prominence of the 510 nm characteristic peak, with fluctuating absorbance values in varying samples collected from each participant (n = 19). The characteristic peak is associated with the reaction between the blue tetrazolium dye-reagent mixture and the cortisol present in the saliva sample. A peak at 510 nm corresponds with the absorbance of the colour green, which is complementary to the magenta colour that is observed within 10 min of the colorimetric reaction taking place (Fig. 5). The absorbance at 510 nm changes as a function of cortisol concentration, which is further reinforced by the increased intensity of the magenta colour seen in samples of higher cortisol concentration, validated by the cortisol ELISA protocol. The peak absorbance values for each spectrum were implemented into the linear regression model to yield predicted cortisol concentration values for the collected saliva samples, presented in Fig. 4. The second derivative spectra were obtained for all samples, to further enhance the spectral features at 510 nm. The second derivative spectrum is often used to quantify the absorption of sample for subsequent determination of the concentration of the target analyte), which is cortisol in this case. The peak separation in the second derivate spectra of all samples further emphasises the linearity of the relationship between blue tetrazolium and cortisol. Thus, reiterating the feasibility of utilising this method towards point-of-care applications in cortisol monitoring. Through the spectral analysis of the collected data, the salivary cortisol concentration for each participant undergoing the MAST protocol was mapped in Figs. 6 and 7 for simpler observation of the trajectory of cortisol levels from baseline measurements (T-Base, point of stress elicitation (T-0) to stress recovery (T-20) and intervention (T-Relax).

### Analysis of salivary cortisol concentrations during MAST protocol via BT and ELISA methods

Fluctuations in salivary cortisol concentrations throughout the duration of the MAST protocol amongst all participants’ samples are presented in Figs. 6 and 7. Evidently, the salivary cortisol concentration values obtained from the prediction model devised from the BT method shows clear likeness to the known cortisol concentration values obtained from the ELISAS. Thus, there is confidence in the feasibility of employing the BT method for salivary cortisol determination within line of the existing gold standard protocols that are utilised by commercial laboratories. Distinctly, the peak cortisol level for each participant differs vastly throughout the MAST protocol, for example, participant 1 showcasing very high baseline cortisol levels (22 ng/ml) with cortisol levels diminishing upon stress elicitation to 4 ng/ml. Comparatively, the observations from samples collected from participant 10 presents low baseline cortisol concentration (1.6 ng/ml) to a spike in cortisol upon stress elicitation and recovery in stages (T-0, T-20) with concentrations reaching 6 ng/ml. The disparity amongst cortisol concentrations in participants unveils the necessity for further stress/cortisol profiling analysis.

### Statistical comparison and validation of performance of BT protocol versus gold-standard ELISA protocol

As mentioned previously, ELISAs are the gold-standard technique for gold-standard for salivary cortisol measurement. Therefore, all samples were investigated using cortisol ELISA kits to yield optical density results which were analysed to determine cortisol concentration using standard curves for calibration, shown in Fig. 1 for each plate. Predicted cortisol concentration values from the BT method were compared the results from the cortisol ELISA protocol to analyse the validity of the proposed optical method against the clinical standard. To analyse the correlation between the predictive models for cortisol concentration, the Pearson’s correlation coefficient was determined (Table 2). There is strong correlation between the BT method and the ELISA method for cortisol concentration as shown in Table 2 for both plate 1, *r*(*38*) = *0.99*, *p* < *0.0001*, and plate 2, *r*(*34*) = *0.99*, *p* < *0.0001*.

**Table 2:** Statistical analysis of dataset 1 (participants 1–10) and dataset 2 (participants 11–19) via Pearson’s correlation coefficient for analysis of BT method results versus cortisol ELISA protocol results.

Dataset 1	Dataset 2
*P*-value < .0001	*P*-value < .0001
R = 0.9998	R = 0.9985
R^2^ = 0.99717	R^2^ = 0.99717

## Discussion

Several conclusions can be drawn from the findings of this study, such as the application of the proposed method for potential technological advancements in the management of mental health. The current state-of-the-art technology for the use of mental health involves the applications of physiological monitoring for the evaluation of psychological stress e.g., smart watches. Physiological measures of stress can include heart rate variability, electroencephalography (EEG) data, and electrodermal activity. However, critical analysis of these existing monitoring techniques has shown several shortcomings in the true quantification of stress, due to the lack of consideration for the measurement of stress hormones^20^. Cortisol and other stress hormones, such as adrenaline and noradrenaline, are the key logical indicators of stress because of their involvement in the stress response. The stress response, often referred to as the ‘fight or flight’ response is the chain of events that take place in the human body upon stress elicitation i.e., a sudden change which interferes with homeostasis. Previous studies have reported on the direct relationship between cortisol concentration and HPA activity, with increases in cortisol in the body translating to resistance stage of the stress response. Hence, the utilisation of the cortisol response towards the quantification of stress and its relationship with mental health is quintessential for the progression towards innovative solutions for the support and treatment of mental disorders beyond traditional interventions such as interview-based techniques and face-to-face therapy^21^.

The presence of a stressor activates the HPA axis and subsequently leads to the release of cortisol into the bloodstream, with previous findings reporting a peak in saliva is observed approximately 20–30 min after stress elicitation^22,23^. The results from this protocol evidently reflect this trend, with salivary cortisol levels reaching maximum levels in the 3^rd^ measurement (T3). Thus, demonstrating the success of the MAST protocol and the BT method in determination of salivary cortisol levels. The simplicity and cost-effectiveness of colorimetric analysis of cortisol holds great potential towards point-of-care applications. As a standard cortisol ELISA kit costs approximately $450 (approximately $12 per test condition), whilst the total cost of the BT method is approximately $375 ($0.60 per test condition). Therefore, the BT method is approximately 95% more cost effective than the standard ELISA method, for the colorimetric determination of salivary cortisol concentrations. Furthermore, standard cortisol ELISA kits are often performed on large batches of samples at a time, to ensure economical use of ELISA kits and reagents, whilst the BT method can be performed ad-hoc on small batches of samples. This ensures that reagents are not wasted and provide salivary cortisol results in real-time.

The ability to monitor salivary cortisol levels towards psychological stress evaluation would be a major technological advancement for the field of mental health monitoring, which remains antiquated to this day by subjective interview-based tools. The aim of this study was to investigate the feasibility of using colorimetric and optical spectroscopy-based techniques towards the determination of salivary cortisol levels with precision and high accuracy that matches the existing gold-standard methodology (ELISA). The results of this current study demonstrate the robustness of the BT method in participants undergoing the MAST protocol and serves as a foundation for the development of spectrophotometric sensor-based technology which exploits the reaction of the blue tetrazolium dye and tetramethylammonium reagents for the detection of precise levels of cortisol in saliva samples.

Findings from the current study showcase the successful performance of the spectrophotometric determination of salivary cortisol with remarkable accuracy of 99.7%, compared to ELISA methods. The proposed optical method showcases linearly correlated absorbance values with cortisol concentration present in the human saliva samples, which can be employed towards optimal stress monitoring. it is evident that the proposed method of utilising blue tetrazolium for cortisol concentration determination is in line with measurements from the current gold standard ELISA method. The high accuracy of the linear prediction model further reiterates the feasibility of employing this technology for accurate psychological stress monitoring (depicted by the Pearson’s correlation coefficient in Table 2. Following comparison and validation of the proposed methodology against the cortisol ELISA protocol, it is apparent that the chromogenic-based optical spectroscopy technique offers lucrative mapping of the participants’ stress activity in response to the MAST, which is reinforced by the subjective stress scores provided by participants post-MAST.

Evidently, existing technologies towards salivary cortisol determination have proven to be highly accurate, albeit with several limitations such as reaction time, the need for skilled personnel and highly specialised instrumentation. Therefore, the accessibility of such technologies e.g., ELISAs and LC–MS/MS for routine use is vastly uncommon, hindered further by the cost of tests. Previous studies have noted the success of the tetrazolium protocol for cortisol determination in plasma and sweat^14,24^. However, salivary cortisol has been found to be directly proportional to serum unbound cortisol and a better measure of adrenal cortical function than serum cortisol^11^. Consequently, the invasiveness of serum cortisol determination towards psychological evaluation can be an unnecessary measure which could potentially lead to further stresses for participants. Although, the use of sweat cortisol has emerged as a desirable measure in recent studies involving stress monitoring, the complexity of HPA activity and its relationship with sweat production can lead to divisive results, especially if participant fitness levels and nutrition are considered. Thereby, salivary cortisol measurements are regarded as the gold standard biomarker of stress, due to its valuable non-invasive mapping of HPA activity and direct proportionality to blood cortisol levels^6,25,26^.

Notably, the complexities between mental health complications and psychological stress processing are evident in this investigation. From the observed post-MAST cortisol concentrations (T-0, T-20), it can be hypothesised that participants that had higher depression inventory scores experienced the stressors of the MAST contrastingly when compared to those who scored lower in the NHS self-assessment (See Supplementary Table S1 online). Participants with higher depression inventory scores had lower cortisol concentrations post-MAST, which could suggest inactivity of the HPA axis. Previous investigations which observed cortisol concentrations in response to stressors showcase that blunted cortisol responses can be indicative of mental illnesses such as clinical depression^27^. The prevalence of this phenomenon in the current study suggests the necessity for future studies which focus on the optimisation of the investigated methodology towards personalised mental health monitoring, aided by adaptive cortisol profiling. Further investigations involving larger volunteer trials that include varied stress-elicitation methods should be considered towards the development of a complete cortisol monitoring system. Whereby, data from long-term routine measurement of salivary cortisol can be utilised in the development of a predictive model for individualised psychological stress evaluation.

Future work in this field will also focus on the optimisation of the spectroscopic technique towards the development of a point-of-care portable monitoring device which can readily analyse salivary cortisol levels for personal mental health monitoring. Most existing mental health monitoring devices utilise physiological signal processing towards stress detection. However, it has been reported that such methods may lead to several inaccuracies in psychological stress evaluation. Primarily due to lack of consideration of the implications of cortisol and other stress hormones on the HPA axis. Evidently, the significance of cortisol is unprecedented in stress evaluation, although the introduction of complimentary stress hormones such as adrenaline, or antagonistic hormones like dehydroepiandrosterone (DHEA) would further consolidate the hormonal determination of stress^21^. Thus, the development of healthcare devices that account for both, physiological and biochemical attributes of psychological stress, would lead to more robust applications of stress monitoring.

It is also essential to note the broader significance of characterising cortisol through colorimetric and optical methods such as those highlighted in the current study. Although cortisol is primarily associated with stress, the excessive production of cortisol in the body or hypercortisolism is known as Cushing’s syndrome. In some cases, Cushing’s syndrome is present in patients who have taken steroid medications, such as steroid tables for a prolonged period of time. Untreated Cushing’s syndrome is associated with extremely poor prognosis, with an estimated 5-year survival rate of 50%^28^. Therefore, in patients suffering from or at risk of this disease due to steroid treatment, the monitoring of cortisol levels is absolutely essential. One of the commonly administered tests for Cushing’s syndrome is a late-night/midnight salivary cortisol test to examine cortisol levels using ELISAs^29^. The introduction of the BT method alongside a point-of-care device for this test could allow for ease of examination and improve patient adherence as tests could be conducted at home, without the need for sending off samples to an external laboratory facility, significantly reducing the costs associated with the procedure. Therefore, the use of the BT method in patients undergoing steroidal treatment could be a pivotal in management of treatment and diagnosis of Cushing’s syndrome.

## Conclusion

In conclusion, the current study has demonstrated the successful implementation of the BT method for rapid and accurate determination of cortisol levels in saliva samples collected from participants undergoing the MAST protocol. With an R^2^ value of 0.997, when compared to ELISAs, the proposed method can be considered as a viable alternative to existing time-consuming and expensive methods for cortisol analysis. This will provide significant advancement in the development of a miniaturised point-of-care device for stress monitoring. The utilisation of such a device on a routine basis will empower individuals to effectively monitor their psychological stress profiles and seek appropriate therapeutic intervention, which is of utmost significance in a growing population of mental illness prevalence.

## Supplementary Information

Supplementary Table S1.

## Data Availability

The authors confirm that the data supporting the findings of this study are available within the article and its Supplementary material. Raw data that support the findings of this study are available from the corresponding author, upon reasonable request.
