# Supplementary material for: Rapid optical determination of salivary cortisol responses in individuals undergoing physiological and psychological stress
Source: Sci Rep. 2024 Dec 30;14:31578. doi: 10.1038/s41598-024-69466-5 (PMC11685491; doi:10.1038/s41598-024-69466-5)
Supplement: Supplementary file 1 — Supplementary Table S1. [file 41598_2024_69466_MOESM1_ESM.docx]

Table S1 - NHS Self-Assessment Depression and Anxiety Inventory Scores, alongside subjective stress rating for each participant.

| **Participant** | **Depression Score** | **Anxiety Score** | **Stress Rating** |
| --- | --- | --- | --- |
| P1 | 4 | 5 | 4.5 |
| P2 | 0 | 0 | 4 |
| P3 | 8 | 4 | 2.5 |
| P4 | 0 | 0 | 3 |
| P5 | 0 | 1 | 4 |
| P6 | 9 | 8 | 2.5 |
| P7 | 4 | 1 | 4 |
| P8 | 2 | 4 | 4 |
| P9 | 8 | 7 | 3.5 |
| P10 | 11 | 11 | 3.5 |
| P11 | 4 | 3 | 2.5 |
| P12 | 5 | 3 | 4 |
| P13 | 1 | 1 | 1.5 |
| P14 | 7 | 9 | 3.5 |
| P15 | 0 | 1 | 3 |
| P16 | 2 | 0 | 1 |
| P17 | 5 | 3 | 3 |
| P18 | 1 | 2 | 3 |
| P19 | 0 | 0 | 2 |
